# Bloodletting Puncture at Hand Twelve *Jing*-Well Points Improves Neurological Recovery by Ameliorating Acute Traumatic Brain Injury-Induced Coagulopathy in Mice

**DOI:** 10.3389/fnins.2020.00403

**Published:** 2020-06-05

**Authors:** Bo Li, Xiu Zhou, Tai-Long Yi, Zhong-Wei Xu, Ding-Wei Peng, Yi Guo, Yong-Ming Guo, Yu-Lin Cao, Lei Zhu, Sai Zhang, Shi-Xiang Cheng

**Affiliations:** ^1^Tianjin Key Laboratory of Neurotrauma Repair, Institute of Neurotrauma Repair of Characteristic Medical Center of Chinese People’s Armed Police Force (PAP), Tianjin, China; ^2^Acupuncture Research Center, Tianjin University of Traditional Chinese Medicine, Tianjin, China; ^3^Central Laboratory of Logistics University of Chinese People’s Armed Police Force (PAP), Tianjin, China; ^4^Zhenxigu Medical Research Center, Beijing, China; ^5^Department of Spine Surgery, Xi’an Honghui Hospital, Xi’an Jiaotong University, Xi’an, China

**Keywords:** traumatic brain injury, coagulopathy, bloodletting puncture at hand twelve *Jing*-well points, controlled cortical impact, model, mouse

## Abstract

Traumatic brain injury (TBI) contributes to hypocoagulopathy associated with prolonged bleeding and hemorrhagic progression. Bloodletting puncture therapy at hand twelve *Jing*-well points (BL-HTWP) has been applied as a first aid measure in various emergent neurological diseases, but the detailed mechanisms of the modulation between the central nervous system and systemic circulation after acute TBI in rodents remain unclear. To investigate whether BL-HTWP stimulation modulates hypocoagulable state and exerts neuroprotective effect, experimental TBI model of mice was produced by the controlled cortical impactor (CCI), and treatment with BL-HTWP was immediately made after CCI. Then, the effects of BL-HTWP on the neurological function, cerebral perfusion state, coagulable state, and cerebrovascular histopathology post-acute TBI were determined, respectively. Results showed that BL-HTWP treatment attenuated cerebral hypoperfusion and improve neurological recovery post-acute TBI. Furthermore, BL-HTWP stimulation reversed acute TBI-induced hypocoagulable state, reduced vasogenic edema and cytotoxic edema by regulating multiple hallmarks of coagulopathy in TBI. Therefore, we conclude for the first time that hypocoagulopathic state occurs after acute experimental TBI, and the neuroprotective effect of BL-HTWP relies on, at least in part, the modulation of hypocoagulable state. BL-HTWP therapy may be a promising strategy for acute severe TBI in the future.

## Introduction

Traumatic brain injury (TBI) is defined as an altered brain function or other evidence of brain pathology caused by an external force. In 2016, there were 27.08 million new cases of TBI, with age-standardized incidence rates of 369 per 100,000 populations (GBD 2016 Traumatic Brain Injury and Spinal Cord Injury Collaborators, 2019). In China alone, age-standardized incidence rates increased by 33.1% for TBI from 1990 to 2016, and China had more patients with TBI than most other countries in the world, causing a huge burden to families and society (GBD 2016 Traumatic Brain Injury and Spinal Cord Injury Collaborators, 2019; Jiang et al., 2019). In most cases of TBI, the brain tissue may be damaged, the blood vessels can rupture and cause bleeding, or a combination of these injuries may occur (Silverberg et al., 2019). Therefore, developing the effective early diagnostic methods and treatment strategies in preventing the diverse and complex nature of the pathological processes activated by TBI poses a major challenge for neurological researchers (Cheng et al., 2018). However, the hypocoagulable state after acute TBI involves complex and multifactorial molecular mechanisms that are poorly understood.

Until now, it has been generally assumed that trauma-induced coagulopathy (TIC) is strongly associated with poorer outcomes and progression of intracranial injury after TBI (Zhang et al., 2015; Chang et al., 2016), and the number of patients with TBI and coagulopathy doubles within 24 h after injury (Greuters et al., 2011). Clinical studies have demonstrated that coagulopathy occurred in nearly two-thirds of severe TBI patients, resulting in hypocoagulopathy associated with prolonged bleeding and hemorrhagic progression after TBI (Harhangi et al., 2008; Laroche et al., 2012; Maegele et al., 2017). It has been shown that TIC contributes to immediate or progressive hemorrhagic contusions in various pathophysiological conditions, including tissue and/or vessel injury, blood–brain barrier disruption, endothelial activation, and inflammation. However, these pathological processes affect the prognosis of TBI. Whether it is possible to improve the prognosis of TBI by altering these pathological processes remains to be elucidated. Additionally, the degree to which these coagulation abnormalities affect neurorestoratology after TBI and whether they are modifiable risk factors for neurorestorative strategies are not known (McCully and Schreiber, 2013; Huang et al., 2018, 2019). Furthermore, coagulopathy is also a common occurrence after severe systemic trauma in the absence of TBI (Spahn et al., 2019), and whether systemic management approaches in these patients might also apply to patients with TBI is unclear. Given these limitations, there is an urgent need to deeply explore the TBI-induced coagulopathic processes and focus primarily on novel treatment of coagulopathy to best manage these TBI patients.

As a treatment of traditional Chinese medicine, acupuncture mechanically inserts fine needles into specific acupoints in the body to stimulate a desired physiologic response, and bloodletting puncture therapy has been applied as one of the first aid measures in various types of emergency for more than 3,000 years in ancient China (Cybularz et al., 2015). With the growing acceptance and application since its endorsement by the National Institutes of Health (NIH), acupuncture has been shown to be safe and effective across a variety of clinical settings (National Institutes of Health, 1998). In particular, bloodletting puncture at hand twelve *Jing*-well points (BL-HTWP), which is a main concept in traditional Chinese acupuncture theory, has been applied to treat various neurological diseases. Our previous study, as well as those from other groups have demonstrated that BL-HTWP could ameliorate blood–brain barrier dysfunction after acute severe TBI (Tu et al., 2016), alleviated cerebral edema in ischemic stroke (Yu et al., 2017), and improved consciousness disorders of acute carbon monoxide poisoning patients (Yue et al., 2015), suggesting that BL-HTWP might be a neuroprotective effect in the brain as a novel approach to treatment of TBI. Nevertheless, the mechanism of this neuroprotective effect is unclear. However, one of the possible mechanism is related to some special ways called meridians (Xu et al., 2014), which connect between the CNS and systemic circulation.

We hypothesized that stimulation of BL-HTWP modulates hypocoagulable state after acute TBI and may exert a neuroprotective effect. Hence, in the present study, the therapeutic efficacy of BL-HTWP against cerebral hypoperfusion, neurological deficits, blood–brain barrier disruption, and vasogenic/cytotoxic edema was evaluated in TBI mice, respectively, and the effects of BL-HTWP on multiple hallmarks of coagulopathy in TBI were investigated.

## Materials and Methods

### Study Design of Experimental Animals

A total of 130 male C57BL/6J mice aged 10 weeks (weighing 20–25 g; Laboratory Animal Center of Academy of Military Medical Sciences, China) were used for this study. All animal procedures were approved by the Institutional Animal Care and Use Committee at the Characteristic Medical Center of PAP. Mice were randomly divided into three groups for assessment: sham-operated group served as uninjured control (Sham), controlled cortical impact (CCI) injury group, and bloodletting puncture at hand twelve *Jing*-well points group (BL-HTWP, abbreviated as BL). Both CCI and BL groups were subjected to CCI insults, and the sham animals underwent all identical surgical craniotomy procedures without receiving CCI.

**FIGURE 1:**
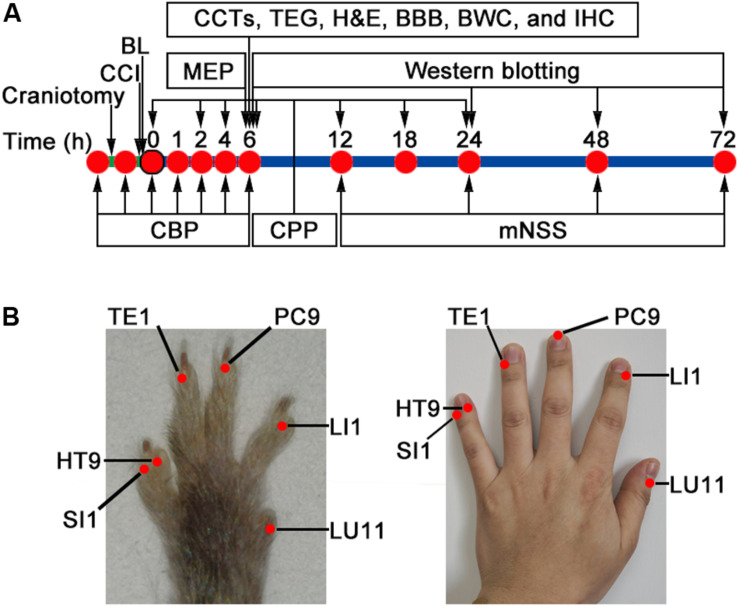
Study design and bloodletting puncture at hand twelve *Jing*-well points (BL-HTWP) treatment in mice. **(A)** Schematic of workflow for experiments. CCI, controlled cortical impact injury; BL, BL-HTWP; CBP, cerebral blood perfusion; CPP, cerebral perfusion pressure; MEP, motor-evoked potential; mNSS, modified neurological severity score; CCTs, conventional coagulation tests; TEG, thromboelastography; BBB, brain-blood barrier permeability; and BWC, brain water content assay. **(B)** After CCI, mice received immediate treatment of BL-HTWP once. BL-HTWP was located at the tips of the mouse toes (*left*), corresponding to the human fingers (*right*), including *Shaoshang* (LU11), *Shangyang* (LI1), *Zhongchong* (PC9), *Guanchong* (TE1), *Shaochong* (HT9), and *Shaoze* (SI1).

All groups were sutured and disinfected after operation. The CCI and Sham groups had only the wound disinfected without any treatment. The study design is presented in Figure 1A, which consisted of four stages: (1) Cerebral blood perfusion (0, 1, 2, 4, 6 h), cerebral perfusion pressure (0, 2, 4, 6, 12, 18, 24 h), motor-evoked potential (6 h), and modified neurological severity score (12, 24, 48, 72 h) post-CCI were performed instantaneously (*n* = 12/group). (2) Six hours after CCI, blood samples were collected for the detection of conventional coagulation tests (*n* = 5/group) and thromboelastography (*n* = 5/group). (3) Six hours after CCI, mice were sacrificed for the measurement of the following parameters: histology, blood–brain barrier, immunofluorescence histochemistry, and brain water content (*n* = 4/group, respectively), and (4) Western blotting was detected at 6, 24, 48, and 72 h post-CCI (*n* = 4 for the Sham group; *n* = 12 for the CCI and BL groups, respectively).

### Controlled Cortical Impact Injury (CCI)

The experimental TBI method used in this study was performed using a CCI protocol as described previously with minor modifications (Cheng et al., 2018). Briefly, mice were placed in the prone position and anesthetized with a continuous flow of isoflurane; then, the skull was exposed by a midline scalp incision. A 4 mm diamter circular craniotomy was made over the right parietal cortex between bregma and lambda with the medial edge 0.5 mm lateral to the midline. TBI mice were then subjected to a CCI injury with an electric cortical contusion impactor device (eCCI-6.3; Custom Design & Fabrication, Richmond, VA, United States) generated at 5 m/s velocity, 1.5 mm impact depth, and a dwell time of 120 ms.

### Bloodletting Puncture at Hand Twelve *Jing*-Well Points (BL-HTWP)

Treatment of BL-HTWP was performed according to previous studies in rats (Tu et al., 2016; Yu et al., 2017), and comparative anatomy was used for point selection, with reference to human anatomical acupoints (Yue et al., 2015) shown in Figure 1B. Briefly, a 21-gauge blood lancet (Hanaco Medical Co., Tianjin, China) was perpendicularly inserted into the skin to a depth of 1 mm in the distal ends of the bilateral acupoints for bloodletting immediately after CCI, with the sequence of LU11 (Shaoshang), LI1 (Shangyang), PC9 (Zhongchong), TE1 (Guanchong), HT9 (Shaochong), and SI1 (Shaoze). Each acupoint was squeezed three to five times to bleed about 5 μl before compressing with cotton balls. BL-HTWP was operated once during the experiment.

### Cerebral Blood Perfusion (CBP)

**FIGURE 2:**
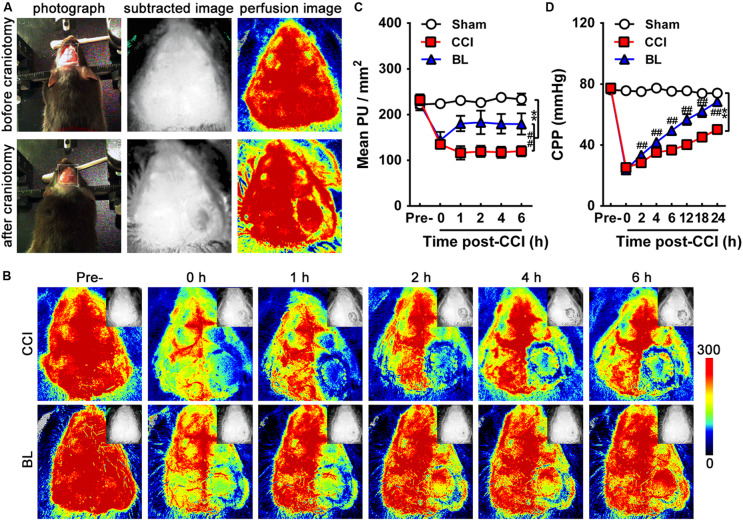
Acute effects of BL-HTWP on cerebral blood perfusion (CBP) and cerebral perfusion pressure (CPP) after TBI. **(A)** CBP was assessed using a blood perfusion imager (PeriCam PSI System) based on laser speckle contrast imaging (LSCI) technology. The dynamic and spatial distribution of CBP was recorded before/after craniotomy, which was performed over the right parietal cortex (photograph, subtracted image, and perfusion image). **(B)** Representative LSCI images in mouse brain at pre-injury, 0, 1, 2, 4, and 6 h post-CCI. Images show areas of yellow-red as high blood perfusion and that of blue-black as low blood perfusion. **(C)** Statistical analysis of mean perfusion units (PU) per square millimeter. **(D)** CPP was assessed at pre-injury, 0, 2, 4, 6, 12, 18, and 24 h post-CCI. Quantitative analysis (*n* = 12) were conducted by one-way ANOVA. **p < 0.01 vs. Sham, ^##^*p* < 0.01 vs. CCI.

CBP was assessed using a blood perfusion imager (PeriCam PSI System, Perimed AB, Stockholm, Sweden) based on laser speckle contrast imaging (LSCI) technology. Briefly, the skull of the anesthetized mouse was exposed by creating a midline skin incision, and the through-skull cerebral blood flow was detected with a speckle contrast. The dynamic and spatial distribution of CBP was then recorded from mice before/after craniotomy (Figure 2A), immediately following CCI (0 h), as well as at 1, 2, 4, and 6 h post-CCI by PSI scanning. The unit of CBP is expressed in PU (perfusion units, PU).

### Cerebral Perfusion Pressure (CPP)

CPP is the difference between mean arterial pressure and intracranial pressure (MAP-ICP) and has become a commonly cited goal in the management of severe TBI. The MAP and ICP of CPP was then recorded from mice before/after craniotomy (Figure 2A), immediately following CCI (0 h), as well as at 2, 4, 6, 12, 18, and 24 h post-CCI by 1 Fr MILRO-TIP transducer (Millar, Houston, TX, United States). Mean arterial pressure was continuously monitored and recorded via the femoral arterial catheter at baseline following CCI. Heart rate was similarly monitored and recorded. Using medical ear–brain glue, the optical fiber probe with a diameter of 0.5 mm was fixed on the bone window opened during modeling to detect ICP.

### Motor-Evoked Potential (MEP)

MEP is used to evaluate the motor nerve function after TBI, including the overall synchronization and integrity of the conduction pathway from the cortex to the muscle by recording the motor compound potential of the cortex in the contralateral target muscle. Measurements of the MEP for the hindlimbs were made using an evoked potential (EP) instrument Viking Quest (Thermo Nicolet Corporation, United States) after 6 h post-CCI. A needle electrode was inserted into the belly of the tibialis anterior muscle as an active electrode, and two needle recording electrodes were inserted into the tendon of the tibialis anterior muscle of both hindlimbs. The reference electrode was placed subcutaneously. The stimulus intensity was 20 V, the stimulation pulse width was 0.5 ms, and the stimulation frequency was 1 Hz. The ground wire was positioned at the tail. Amplitude (mV) was the peak-to-peak voltage, meaning the potential difference between the most positive and the most negative peaks, and latency (ms) was time between the start of the stimulus to the appearance of the initial response wave.

### Modified Neurological Severity Score (mNSS)

mNSS is an independent set of scales, which was performed to evaluate the post-injury neurological functions of mice (*n* = 12/group) before and 12, 24, 48, and 72 h after CCI, which is a composite of motor, sensory, balance, and reflex tests with the maximum scores of 6, 2, 6, and 4 points, respectively (Wang et al., 2014). Thus, higher scores indicate more severe neurological dysfunction (13–18 = severe, 7–12 = moderate, and 1–6 = mild).

### Preparation and Collection of Blood Samples

After 6 h of CCI, blood samples (∼1 ml/mice) were collected via direct left ventricle puncture. Blood for the laboratory analysis, including CCTs and TEG assays, was collected in a vacuum tube containing 0.109 M (3.2%) of sodium citrate. The ratio of sodium citrate to whole blood is 9:1. The vacuum tube was inverted eight times to allow the blood to mix with sodium citrate to prevent clotting.

### Conventional Coagulation Tests

Citrated plasma tubes were prepared after centrifugation at room temperature for 10 min at 3,000 × *g*. Then, CCTs – hematology parameters including prothrombin time (PT), activated partial thromboplastin time (aPTT), international normalized ratio (INR), plasma fibrinogen (FIB), thrombin time (TT), prothrombin time activity (PTA), platelet counts, and coagulation Factor VII and VIII levels—were measured with the STA-R evolution coagulation analyzer (Diagnostica Stago S.A.S, Asnières sur Seine, France) in accordance with the laboratory standard operating procedure.

### Thromboelastography (TEG)

TEG assay was performed with non-activated citrated whole blood using a TEG 5000 System (Haemoscope Corporation, Niles, IL, United States) in accordance with the manufacturer’ instructions. Six hours after CCI, samples were tested at room temperature until all of the following parameters were recorded: reaction time [R, minutes (time to first clot formation)], clotting time [K, minutes (time until the clot reaches a fixed strength)], angle [α, degrees (rate of clot formation)], and maximum amplitude [MA, millimeters (maximum strength of the platelet-fibrin clot)].

### Histological Analysis

Six hours after CCI, mice were subjected to cardiac perfusion with 0.1 M PBS followed by fixation with 4% polyoxymethylene. The brains were removed and postfixed in 4% polyoxymethylene solution for 12 h, and tissues were frozen and embedded in Tissue-Tek OCT. Frozen tissues were cryosectioned at a thickness of 20 μm. The brain tissue sections with the edge of injury were taken, and 10 slices were taken every 1 mm. Representative sections from each mouse were stained with cresyl violet (CV) to assess the neurological damage, or stained by hematoxylin and eosin (*H&E*) to evaluate cerebrovascular histopathological changes. Coronal sections that spanned across the contusion areas were stained with CV (Sigma-Aldrich, St. Louis, MO, United States) as described previously (Cheng et al., 2018). Slice was stained with standard H&E staining to determine the presence of morphological abnormalities and microbleedings post-CCI.

### Permeability of Brain–Blood Barrier

After 5 h of CCI, mice were administered 200 μl of Evans blue (EB; 2% *w/v*; i.p.; Sigma-Aldrich, St. Louis, MO, United States), allowed to circulate for 1 h prior to sacrifice as described previously (Main et al., 2018). Mice were then perfused transcardially with saline to remove intravascular EB dye, and the right hemispheres were immediately removed and separated along the midline. Each sample was weighed, homogenized, and incubated at room temperature for 24 h. Then, the supernatant was collected, and absorbance was measured at 620 nm after centrifugation.

### Brain Water Content

Cerebral edema was evaluated by water content of the brain tissue at 6 h post-CCI with the wet-to-dry weight ratio (Genet et al., 2017). Following sacrifice, the cerebellum and bilateral olfactory bulbs were discarded, and the brain tissue was separated into the left and right hemispheres along the midline. The right hemisphere was taken, the surface water was dried, and the brain tissue was placed on an electronic balance to weigh it and then dried for 24 h at 110°C in an oven, and re-weighed to obtain the dry weight. Brain water content was calculated: BWC = (wet weight - dry weight)/wet weight × 100%.

### Immunofluorescence Histochemistry

Six hours after CCI, mice were subjected to cardiac perfusion with 0.1 M PBS followed by fixation with 4% polyoxymethylene. The brains were removed and postfixed in 4% polyoxymethylene solution for 12 h, and tissues were frozen and embedded in Tissue-Tek OCT. Frozen tissues were cryosectioned at a thickness of 20 μm. The brain tissue sections with the edge of injury were taken, and 10 slices were taken every 1 mm. Brain tissue sections were blocked with 10% bovine serum albumin (BSA) for 1 h, then incubated overnight at 4°C with primary antibodies: rabbit anti-neuronal nuclei (NeuN; ab104225; 1:500; Abcam), anti-glial fibrillary acidic protein (GFAP; #12389S; 1:200; Cell Signaling Technology), and anti-ionized calcium-binding adapter molecule 1 (Iba-1; 10904-1-AP; 1:400; Proteintech). After washing, slides were stained with the appropriate secondary antibodies for 30 min in darkness at 37°C. DAPI was then used to stain the nuclei. Under 400 times fluorescence microscope (Leica DMI400B; Germany), the hippocampus on the injured side was taken as the observation area, four fields from the peritraumatic area in each section were imaged, and the percentage of NeuN + -, GFAP + -, and Iba-1 + - cells were counted using Image J software. Finally, the average number of positive cells in the four sections was calculated as the number of positive cells in the tissue section. All analyses were performed in a blinded manner.

### Western Blotting

Western blotting analysis was performed to detect the protein expression levels of tight junction proteins (Occludin and ZO-1), water channel protein aquaporin 4 (AQP4), inflammatory-related hallmarks including interleukin-6 (IL-6), IL-1β, intercellular adhesion molecule 1 (ICAM-1), and hypoxia-inducible factor-1α (HIF-1α), and trophic factors such as brain-derived neurotrophic factor (BDNF) and vascular endothelial growth factor (VEGF), respectively. Mice were sacrificed, and their brains were rapidly removed at 6, 24, 48, and 72 h post-CCI. For protein extraction, injured brain tissues (10 mg) were isolated and lysed in 4% SDS buffer then homogenized on ice. The proteins were recovered by centrifugation at 13,000 rpm for 15 min at 4°C. Protein samples of each group were separated by 10% polyacrylamide gel electrophoresis and transferred onto nitrocellulose membranes, which were blocked by 10% non-fat milk and incubated at a dilution of 1:1,000 overnight at 4°C with the primary antibodies as follows: Occludin (ab167161; Abcam), ZO-1 (21773-1-AP; Proteintech), AQP4 (ab46182; Abcam), IL-6 (BS6419; Bioworld), IL-1β (BS6067; Bioworld), ICAM-1 (BS7138; Bioworld), HIF-1α (ab179483; Abcam), BDNF (BS6533; Bioworld), VEGF (BS6496; Bioworld), and GAPDH (MB001; Bioworld). After washing, membranes were incubated at room temperature for 2 h with secondary antibody (IgG at 1:10,000 dilutions; KPL). Protein was developed with ECL reagent and visualized using an Amersham Imager 600 (GH Healthcare, Pittsburgh, PA, United States). The density of bands was determined by Scion Image 4.0 software. GAPDH was used as internal control. All analyses were performed in a blinded manner.

### Statistical Analysis

All data were presented as means ± standard deviation (SD). SPSS 16.0 (IBM, Armonk, NY, United States) was used for analysis. Statistical analyses were performed using one-way analysis of variance (ANOVA) followed by *post hoc* comparison. All tests were two-tailed, and a value of *p* < 0.05 was considered to be statistically significant.

## Results

### BL-HTWP Attenuates Cerebral Hypoperfusion Post-acute TBI

To evaluate the acute effects of the BL-HTWP on cerebral blood perfusion (CBP) after TBI, TBI was induced by controlled cortical impact (CCI) leading to cortical lesions with reduced CBP. We showed representative high-resolution CBP images with dynamic changes at pre-injury, immediately following CCI (0 h), as well as 1, 2, 4, and 6 h post-CCI in Figure 2B. As shown in Figure 2C, the mean CBP values determined before CCI were similar among the Sham, CCI, and BL mice. Immediately after CCI, a dramatic decrease in CBP occurred in the CCI and BL groups (134.9 ± 7.7 and 143.90 ± 18.1 PU/mm^2^) compared to that of the Sham mice (223.8 ± 8.9 PU/mm^2^, all *p* < 0.01). The severe reduction in CBP for the CCI group did not significantly improve within 6 h, but cerebral perfusion for the BL-HTWP group was partly recovered after 1 h and consistently remained higher at 2, 4, and 6 h compared to that in CCI mice (BL vs. CCI, 1 h, 180.4 ± 16.8 vs. 116.3 ± 14.7; 2 h, 183.0 ± 25.6 vs. 119.5 ± 12.1; 4 h, 179.8 ± 21.6 vs. 117.7 ± 12. h, 179.5 ± 23.0 vs. 120.0 ± 11.1 PU/mm^2^; all *p* < 0.01). Additionally, CPP in both the CCI and BL groups immediately declined at 0 h post-CCI (25.25 ± 1.87, 23.77 ± 2.96 mmHg) compared with the Sham mice (75.71 ± 1.72 mmHg; all *p* < 0.01). After 24 h, the CPP of mice in the CCI and BL groups increased in varying degrees, but that of BL group consistently remained higher compared to that in CCI mice (all *p* < 0.01) (Figure 2D). These results suggested that BL-HTWP treatment promoted CBP and CPP restoration and their maintenance to attenuate cerebral hypoperfusion after acute TBI.

### BL-HTWP Improves Neurological Recovery Post-acute TBI

**FIGURE 3:**
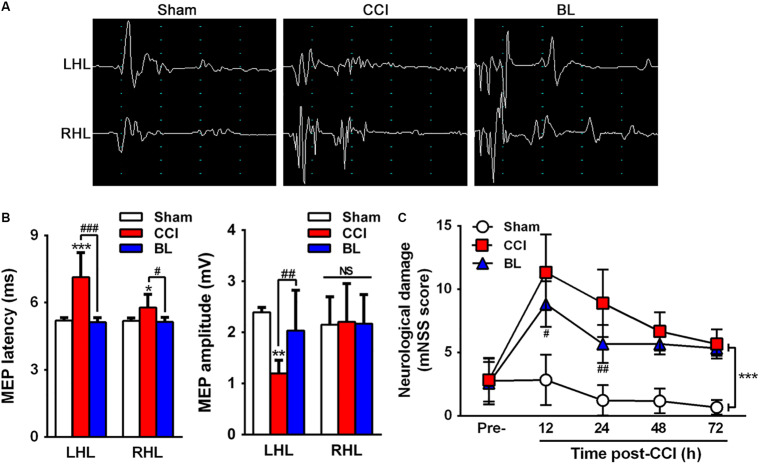
Efficacy of BL-HTWP in neurologic recovery post-TBI. **(A)** Representative images of motor-evoked potential (MEP). LHL, left hindlimb (CCI-contralateral limb); RHL, right hindlimb (CCI-ipsilateral limb). **(B)** Alterations of MEP amplitude and latency in the mice of Sham, CCI, and BL at 6 h after CCI. **(C)** Modified neurological severity (mNSS) score was determined at pre-injury, 12, 24, 48, and 72 h after CCI. Quantitative analysis (*n* = 12) was conducted by one-way ANOVA. *p < 0.05, **p < 0.01 vs. Sham; ^#^*p* < 0.05, ^##^*p* < 0.01,^ ###^*p* < 0.001 vs. CCI. NS indicates *p* > 0.05.

To gain further insight into the efficacy of BL-HTWP in neurological recovery after TBI, motor-evoked potential (MEP) and mNSS tests were performed at 6 h (MEP) (Figure 3A), as well as 12, 24, 28, and 72 h (mNSS) post-TBI. Compared with the Sham group, CCI mice had significantly prolonged latency (7.13 ± 1.10 vs. 5.20 ± 0.12 ms, *p* < 0.01) in the left hindlimb and that (5.78 ± 0.58 vs. 5.18 ± 0.13 ms, *p* < 0.05) in the right hindlimb on the bilateral motor cortex stimulation at 6 h post-TBI (Figure 3B). Also, CCI mice had sharply deduced amplitude (1.20 ± 0.26 vs. 2.39 ± 0.09 mV, *p* < 0.01) in the left hindlimb, but not significantly changed in the right hindlimb. In the BL group, however, the latency in both hindlimbs (left, 5.12 ± 0.20 ms; right, 5.14 ± 0.19 ms, *p* < 0.001 or 0.05) was decreased, and the amplitude in the left hindlimb (2.04 ± 0.79 mV, *p* < 0.01) was increased after BL-HTWP in comparison with the values of the CCI group. Additionally, in contrast to the Sham group, significant neurological deficits were detected in the CCI and BL groups at 12, 24, 48, and 72 h post-TBI (all *p* < 0.01) (Figure 3C). However, the mNSS scores were found to be sharply reduced in BL mice, compared to those in CCI group at 12 h (*p* < 0.05) and 24 h (*p* < 0.01) after TBI. Altogether, these results confirmed that BL-HTWP ameliorated electrophysiological deficit and improved neurological recovery.

### BL-HTW Preverses Acute TBI-Induced Hypocoagulable State and Increases the Level of Procoagulant

**FIGURE 4:**
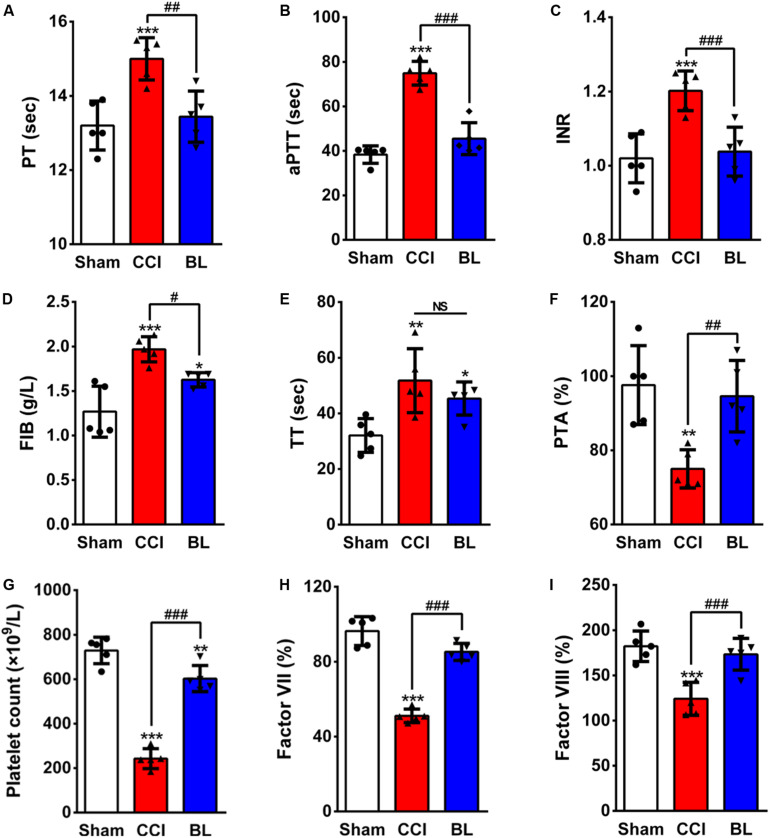
Effects of BL-HTWP on conventional coagulation tests (CCTs) after TBI. At 6 h post-CCI, mice blood samples were assayed for coagulation by the CCT method with prothrombin time (PT, **A**), activated partial thromboplastin time (aPTT, **B**), international normalized ratio (INR, **C**), plasma fibrinogen (FIB, **D**), thrombin time (TT, **E**), prothrombin time activity (PTA, **F**), platelet count **(G)**, Factor VII **(H)**, and Factor VIII **(I)**. Quantitative analysis (*n* = 5) were conducted by one-way ANOVA. *p < 0.05, **p < 0.01, *** *p* < 0.001 vs. Sham; ^#^*p* < 0.05, ^##^*p* < 0.01,^ ###^*p* < 0.001 vs. CCI. NS indicates *p* > 0.05.

Because normal hemostasis depends on a balance between bleeding and thrombosis, this balance can be altered following TBI, leading to coagulopathy. So, we questioned whether BL-HTWP treatment is associated with TBI-induced coagulopathy. We first examined the coagulatory state at 6 h post-TBI using conventional coagulation tests (CCTs). PT reflects tissue factor and exogenous coagulation function. APTT mainly reflects the function of endogenous blood coagulation. The combination of PLT and FIB reflects the ability of thrombosis. When PT and aPTT increased and PLT and FIB decreased, it indicates high DIC risk. Among the most valuable parameters examined in the CCTs assays, the PT and aPTT (15.00 ± 0.57 s, 74.90 ± 5.32 s), INR and TT (1.20 ± 0.05, 51.76 ± 11.51 s), and the FIB values (1.97 ± 0.14 g/L) of the CCI mice were much higher than in the Sham group (PT 13.20 ± 0.66 s, aPTT 38.34 ± 3.90 s, INR 1.02 ± 0.07, TT 32.06 ± 6.05 s, and FIB 1.27 ± 0.29 g/L; *p* < 0.001 or 0.01, respectively) (Figures 4A–E). However, they were sharply lower in the BL mice (PT 13.44 ± 0.69 s, aPTT 45.50 ± 7.17 s, INR 1.04 ± 0.07, and FIB 1.63 ± 0.08 g/L) compared with the CCI group (*p* < 0.001, 0.01, or 0.05). There was no significant difference between the BL group and the CCI group in the TT values (45.35 ± 5.96 s vs. 51.76 ± 11.51 s; *p* > 0.05). Additionally, there was significant shorter PTA in CCI mice (75.00 ± 5.15%) than in the Sham controls (97.60 ± 10.64%; *p* < 0.01) (Figure 4F), but BL significantly prolonged the PTA (94.60 ± 9.66%) compared with the CCI group (*p* < 0.01). Furthermore, BL-HTWP better preserved the platelet counts (603 vs. 243 × 10^9^/L; *p* < 0.001) (Figure 4G) and significantly increased in Factor VII (85% vs. 51%; *p* < 0.001) (Figure 4H) and Factor VIII (173% vs. 124%; *p* < 0.001) (Figure 4I) compared with that of the CCI group, and Factor VII and VIII levels had no statistical difference compared to those of the Sham mice (all *p* > 0.05).

**FIGURE 5:**
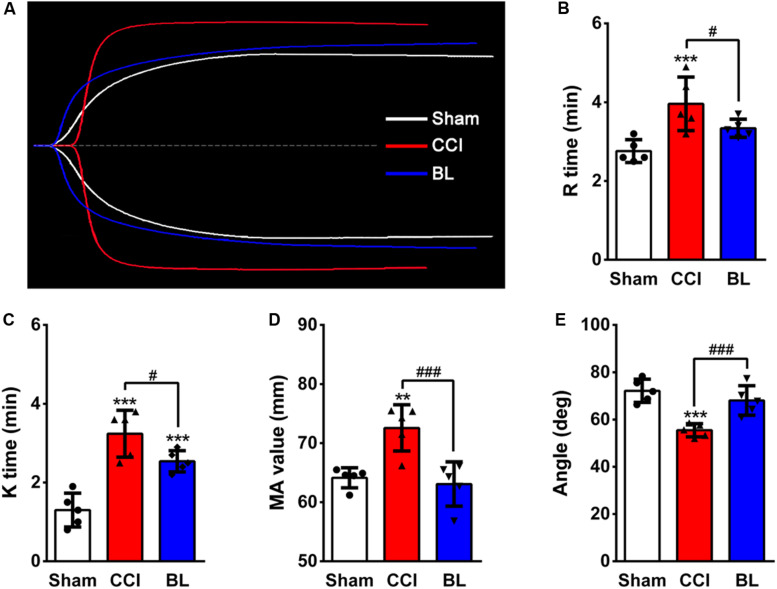
Effects of BL-HTWP on thromboelastography (TEG) values after TBI. **(A)** Representative TEG tracings from the Sham (white line), CCI (red line), and BL (blue line) group. Impact of TEG after bloodletting puncture at HTWP (BL) on R time **(B)**, K time **(C)**, α-angle **(D)**, and MA value **(E)** in mice at 6 h post-CCI. Quantitative analysis (*n* = 5) were conducted by one-way ANOVA. **p < 0.01, ***p < 0.001 vs. Sham; ^#^*p* < 0.05, ^###^*p* < 0.001 vs. CCI.

In addition to CCTs, thromboelastography (TEG) assays were also performed. Representative TEG tracings from the groups are also shown in Figure 5A. Among the parameters examined in the TEG assays, the R time, K time, and MA value of the CCI groups (3.96 ± 0.68 min, 3.24 ± 0.59 min, 72.58 mm) were significantly higher compared with the Sham control (2.76 ± 0.29 min, 1.30 ± 0.43 min, 64.14 ± 1.69 mm; *p* < 0.001 or 0.01) (Figures 5B–D), but the α angle of the CCI group (55.46 ± 2.81°) was sharply lower than that of the Sham group (72.16 ± 4.90°; *p* < 0.001) (Figure 5E). However, the BL mice showed a shorter R (3.34 ± 0.23 min), K (2.54 ± 0.27 min), MA value (63.08 ± 3.74 mm), and a longer α angle (68.06 ± 6.26°) compared with that of the CCI group (*p* < 0.001 or 0.05). Taken together, these results demonstrated that TBI induced a hypocoagulable state with prolonged bleeding at 6 h post-CCI; however, treatment of blood with BL-HTWP resulted in a shorter PT, aPTT, INR, FIB, a lower R, K, MA value, a greater PTA, and a larger angle than CCI alone, indicating that BL-HTWP could reverse acute TBI-induced hypocoagulability.

### BL-HTWP Promotes BBB Integrity to Reduce Vasogenic Edema

**FIGURE 6:**
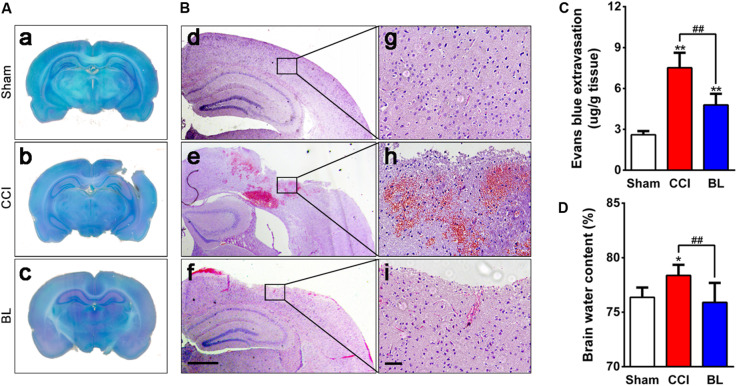
Effects of BL-HTWP on neurological damage and cerebrovascular histopathological changes post-TBI. Brain tissues were collected at 6 h after TBI. **(A)** Lesion assessment was performed on cresyl violet-staining brain sections. **(B)** Microbleedings were observed with hematoxylin and eosin (H&E) staining. (d–f) Representative images of contusion areas visualized with H&E staining. Scale bar, 250 μm. (g–i) Magnification of images shown in (d–f). Scale bar, 50 μm. **(C)** Blood–brain barrier permeability was measured by assessing the extravasation of Evans blue (EB) dye. **(D)** Cerebral edema was evaluated by water content of the brain tissue with the wet-to-dry weight ratio. Quantitative analysis (*n* = 4) were conducted by one-way ANOVA. *p < 0.05, **p < 0.01 vs. Sham; ^##^*p* < 0.01 vs. CCI.

Coagulation abnormalities increased risk for potentially dangerous bleeding and worse outcome after TBI. In this regard, we explored whether BL-HTWP treatment could attenuate progression of intracranial hemorrhagic lesions by reversing hypocoagulability at 6 h after TBI. As expected, BL mice showed smaller cortical lesion sizes than those of CCI animals, and representative examples of cresyl violet-staining sections are shown in Figure 6A. *H&E* examination also confirmed the presence of tissue loss and intracranial hemorrhage at the area surrounding the cortical lesion of the CCI mice, but BL-treated mice displayed smaller hemorrhagic areas (Figure 6B).

After TBI, disruption of cerebral blood vessels – mainly in a blood–brain barrier (BBB) breakdown – results in the progression of hemorrhagic lesions, which underlies brain edema formation. So, the effects of BL-HTWP on BBB permeability and brain edema were observed. Levels of EB extravasation of the injured hemisphere were significantly higher in the CCI group (7.5 ± 1.1 μg/g) compared with those in the Sham control (2.6 ± 0.3 μg/g, *p* < 0.01). However, the extravasation demonstrated lower values in the BL group (4.8 ± 0.8 μg/g) than those in the CCI mice (*p* < 0.01) (Figure 6C). Additionally, CCI mice developed obvious brain edema (78.8 ± 0.5%) compared with the Sham group (76.1 ± 0.8%, *p* < 0.05), whereas the value in the BL mice was lower (76.3 ± 1.0%) than those of the CCI group (*p* < 0.01), and similar to that of the Sham animals (*p* > 0.05) (Figure 6D). Overall, these results suggested that BL-HTWP ameliorated TBI-induced cerebrovascular histopathology including disruption of BBB and vasogenic cerebral edema formation.

### BL-HTWP Reduces the Damage of Gliovascular Unit

**FIGURE 7:**
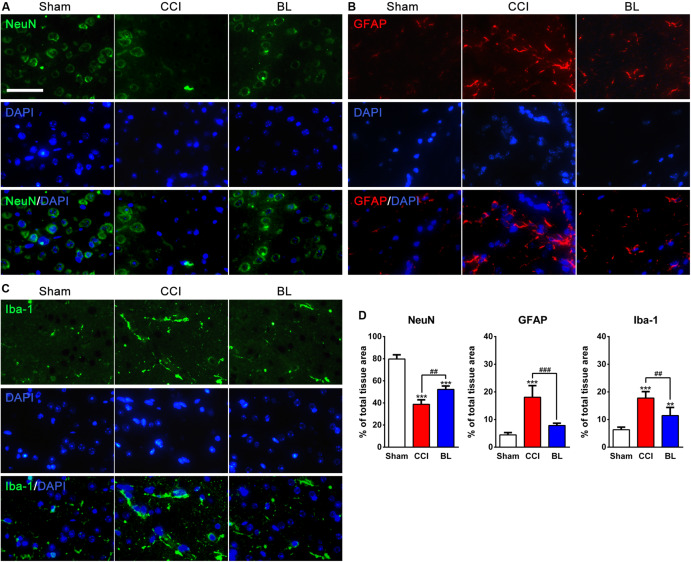
Neuroprotective effects of BL-HTWP against neural death and activation of microglia and astrocytes post-TBI. Brain tissues were collected at 6 h after TBI, and the presence of neural death and microglia or astrocyte activation was visualized by immunofluorescence in the perilesional brain area of mice. **(A–C)** Representative images (green: NeuN or Iba-1, red: GFAP, blue: DAPI). Scale bar, 50 μm. **(D)** Quantification of percentage of NeuN^+^-, GFAP^+^-, and Iba-1^+^-cells of total tissue area, respectively. Quantitative analysis (*n* = 4) was conducted by one-way ANOVA. **p < 0.01, ***p < 0.001 vs. Sham; ^##^*p* < 0.01, ^###^*p* < 0.001 vs. CCI.

In order to evaluate the damage of gliovascular unit generated in the perilesional area of mice after TBI, brain sections were immunostained for NeuN (Figure 7A), GFAP (Figure 7B), and Iba-1 (Figure 7C), which are specific markers of neurons, astrocytes, and microglia, respectively. As shown in Figure 7D, NeuN-positive neurons accounted for approximately 80% in the Sham mice, but this percentage was dramatically decreased at 6 h after TBI, with only approximately 39% of cells expressing NeuN (*p* < 0.001). However, the percentage of NeuN^+^-cells in the BL group was higher (∼52%) than those in the CCI group (*p* < 0.01).

Additionally, In the Sham brains, GFAP and Iba-1 immunoreactivity were relatively weak in the Sham brains. At 6 h post-TBI, the percentage of GFAP^+^- and Iba-1^+^-cells (18.06 ± 4.20% and 17.78 ± 2.33%) were sharply upregulated in the perilesional area compared with those of the Sham group (4.44 ± 0.85% and 6.32 ± 0.92%, all *p* < 0.001). In contrast, fewer GFAP^+^-cells (7.85 ± 0.82%, *p* < 0.001) and Iba-1^+^-cells (11.43 ± 2.96%, *p* < 0.01) were found in the BL group than in the CCI mice.

### BL-HTWP Regulates Multiple Hallmarks of Coagulopathy in TBI

**FIGURE 8:**
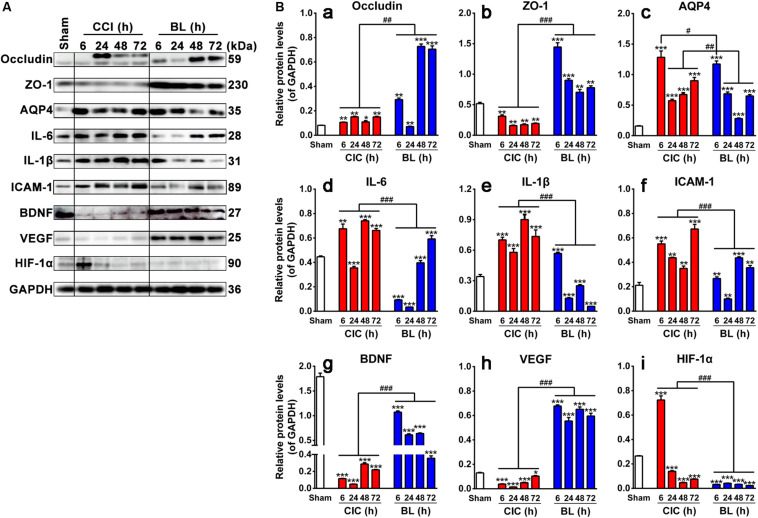
Effects of BL-HTWP on blood–brain barrier, inflammation, and neurogenesis-related hallmarks post-TBI. **(A)** Representative Western blot results for the protein expression at 6, 24, 48, and 72 h after TBI. **(B)** Quantification of relative protein levels of Occludin (a), ZO-1 (b), AQP4 (c), IL-6 (d), IL-1β (e), ICAM-1 (f), BDNF (g), VEGF (h), and HIF-1α (i), respectively. Quantitative analysis (*n* = 4 for Sham group; *n* = 12 for CCI and BL groups, respectively) were conducted by one-way ANOVA. *p < 0.05, **p < 0.01, ***p < 0.001 vs. Sham; ^#^*p* < 0.05, ^##^*p* < 0.01, ^###^*p* < 0.001 vs. CCI.

To identify biochemical alterations induced by TBI and further explore the underlying mechanisms of protective action of BL-HTWP, a time course experiment was performed to evaluate the changes of multiple protein expression levels related to blood–brain barrier integrity, inflammation, and trophic factors at 6, 24, 48, and 72 h after TBI with Western blotting analysis (Figure 8A).

#### Expression States of Occludin, ZO-1, and AQP4

As shown in Figures 8Ba–c, levels of Occludin and ZO-1 were found to be significantly lower in the CCI than in the Sham group at different time points (*p* < 0.001 or 0.01), while levels of those were dramatically increased in the BL compared with those in the CCI group (*p* < 0.001) except for the Occludin levels at 24 h post-TBI. Levels of AQP4, on the other hand, were significantly higher in both the CCI and BL groups than in the Sham group over a broad period (all *p* < 0.001), but BL mice showed lower levels of AQP4 at 6 h (*p* < 0.05) and 24–72 h (all *p* < 0.01) than those of the CCI mice.

#### Expression States of IL-6, IL-1β, and ICAM-1

As shown in Figures 8Bd–f, IL-6, IL-1β, and ICAM-1 levels were strongly elevated in CCI mice above Sham (*p* < 0.001 or 0.01) except for the IL-6 levels at 24 h post-TBI. In contrast, levels of those proteins were dramatically decreased in the BL compared with those in the CCI group (*p* < 0.001).

#### Expression States of BDNF, VEGF, and HIF-1α

As shown in Figures 8Bg–h, levels of BDNF and VEGF levels were reduced spontaneously at 6, 24, 48, and 72 h post-TBI. On the contrary, the expression levels of BDNF and VEGF were always maintained as the higher states in the BL mice than those in the CCI groups (all *p* < 0.001). Interestingly, the level of HIF-1α sharply increased at 6 h and decreased from 24 to 72 h compared with the Sham mice (all *p* < 0.001). However, the elevated levels of HIF-1α were reversed by the administration of BL immediately after TBI, and this low level was maintained over a broad period compared with the CCI group (all *p* < 0.001) (Figure 8Bi).

Overall, these data indicated that BL-HTWP might play a vital role in mediating the upregulation of tight-junction molecules to protect the BBB integrity, downregulation of pro-inflammatory cytokines to attenuate neuroinflammation, or modulation of trophic factors to promote neuroprotection.

## Discussion

In the present study, we demonstrated for the first time that BL-HTWP, a treatment of traditional Chinese medicine, attenuated cerebral hypoperfusion, electrophysiological and neurological deficits in mice after acute TBI. These functions may be associated with amelioration of intracranial hemorrhagic lesions and cerebral edema caused by reversing acute TBI-induced hypocoagulable state. We further revealed that the anti-edematous effect of BL-HTWP may be through alleviating BBB breakdown (vasogenic edema) and attenuating activation of glial cells (cytotoxic edema), indicating that it was necessary for mediating the upregulation of tight-junction molecules to protect the BBB integrity, downregulation of pro-inflammatory cytokines to attenuate neuroinflammation, or modulation of trophic factors to promote neuroprotection.

By all accounts to date, normal hemostasis depends on an intricate balance between bleeding and thrombosis formation, and this balance can be altered after trauma. Conversely, coagulopathic bleeding is extremely difficult to control, resulting in diffuse bleeding involving uninjured sites.

Recently, various studies have highlighted the importance of coagulopathy in isolated TBI subsequently displaying hemorrhagic progression and ongoing intracranial hemorrhage. More than 60% of patients with severe head injury have coagulopathy. Approximately 50% of patients with coagulopathy after TBI exhibit progressive bleeding injury and persistent intracranial hemorrhage within 48 h (Tong et al., 2012; Castellino et al., 2014; Juratli et al., 2014; Yuan et al., 2016; Jenkins et al., 2018; Zhang et al., 2018). Coagulopathy presented a power predictor of outcome and prognosis in TBI, contributing to higher risk of mortality and unfavorable outcome than in TBI without coagulopathy. An evidence-based study of 1,718 sample sizes found that patients with coagulopathy after TBI can have a mortality rate of up to 45.1% (Epstein et al., 2014; Talving et al., 2009). Early correction of coagulation disorders after TBI is independently related to the survival of patients (Epstein et al., 2015). The most common choice for the treatment of coagulation disorders associated with TBI is the transfusions of blood components, including red blood cell transfusions, fresh frozen plasma and platelet-concentrate transfusions (Baharoglu et al., 2016; Boutin et al., 2016; Chang et al., 2017). Besides, there is the treatment of coagulation factor concentrates, such as prothrombin complex concentrate, fibrinogen, recombinant Factor VIIa (Brown et al., 2010; Rourke et al., 2012; Yanamadala et al., 2014), and some drug treatments including hemostatic agents, tranexamic acid, and desmopressin (CRASH-2 Trial Collaborators et al., 2010; Roberts et al., 2013; Naidech et al., 2014; Myles Paul et al., 2017). The above treatments have made some progress in alleviating hypercoagulable state and preventing progressive hemorrhage, but there is no consensus on the strategy for the use of blood transfusion therapy (Washington et al., 2011). The treatment of coagulation factor concentrates and drug therapy also has potential risks (Shen et al., 2015). The reason might be due to the complexity of the cascade system of blood coagulation mechanism. It is difficult to balance the whole system by simply regulating one of a link. Nevertheless, a much complex series of events associated with coagulopathy occurs either simultaneously or sequentially after TBI, and the clinical coagulopathic bleeding course has been considered to reflect rapid progression from a state of hypercoagulability to hypocoagulability (Maegele et al., 2017). Intravascular coagulation can occur within 6 h after TBI, which is characterized by activation of the coagulation cascade system, resulting in fibrin deposition and intravascular microthrombosis, potential disturbance of cerebral microcirculation, and increased consumption of coagulation, leading to further platelet depletion (Chauny et al., 2016). On the other hand, microvessels serve as a circulation platform for initiating and amplifying coagulation, which usually occurs on the surface of activated platelets and locates at the injured site, resulting in non-focal and diffuse clotting. Previous studies have also shown that TBI, itself, does not cause early coagulation disorders, but it must be combined with low perfusion to cause coagulation disorders, and the destruction of the integrity of the cerebrovascular microvascular wall caused by impact will quickly activate the coagulation cascade (Maegele et al., 2017).

As the most commonly used assessment of coagulation abnormalities, conventional coagulation tests (CCTs) and Thromboelastography (TEG) can facilitate prediction of outcome after TBI (Gonzalez et al., 2014; Joseph et al., 2014). Several studies had used the prothrombin time (PT), activated partial thromboplastin time (aPTT), international normalized ratio (INR), or other values for CCTs (MacLeod et al., 2003; Hoyt et al., 2008), as well as reaction time (R time, which means aggregation time of fiber factor), clotting time (K time, which means the time of fibrin formation), maximum thrombus amplitude (MA, reflect the binding ability of platelets to fibrin), and alpha angle values (α values, reflect the rate of fibrinogen coagulation) for TEG to describe coagulopathy after TBI (Davis et al., 2013; Castellino et al., 2014). As expected, the results of our study found decreased coagulation activity at 6 h following isolated TBI, prolonged PT, aPTT, INR, TT, increased FIB, as well as decreased PTA. Furthermore, the increased R, K, and MA values, as well as decreased α angle, also indicate hypocoagulable tendencies. In contrast, BL mice received immediate treatment of BL-HTWP showing recovery of hypocoagulopathy induced by TBI, which was demonstrated by CCTs or TEG variables, such as decreased PT, aPTT, INR, TT, R, K, and MA values, as well as elevated PTA and α angle at 6 h after TBI, suggesting that BL-HTWP could reverse acute TBI-induced hypocoagulable state. Taking current studies into consideration, we further tried to decipher the underlying mechanisms of this anti-hypocoagulopathic effect of BL-HTWP after acute TBI.

After severe trauma and blood loss, the organism is faced with the competing interests of both limiting further blood loss and limiting microvascular thrombosis to maintain end-organ perfusion in a low-flow state (Chang et al., 2016). However, coagulopathy induced by isolated TBI is likely different from that of other trauma owing to, at least in part, the existence of intact blood–brain barrier (BBB), which may play a key role in the response to vessel injury and coagulation. The release of tissue factors (TF) caused by BBB destruction is also considered to be one of the main causes of blood coagulation disorder after TBI and runs through the process of TBI. Within minutes of TBI, due to the destruction of the BBB, a large number of TF enters the peripheral blood and widely binds with VII factors to trigger exogenous coagulation (Hoffman and Monroe, 2009; Genet et al., 2013; Salehi et al., 2017). With the further destruction of the blood–brain barrier after secondary injury, a large number of platelets are consumed, resulting in intracranial hemorrhage and systemic DIC (Castellino et al., 2014; Atefi et al., 2016; Leeper et al., 2019). The human brain contains ∼644 km of blood vessels, which supply neural cells with oxygen, energy, and nutrients from the brain to the systemic circulation (Zlokovic, 2008). BBB is a continuous endothelial membrane within brain microvessels, and endothelial cells are connected by tight junctions, which involve all kinds of proteins such as Occludin and ZO-1 (Sweeney et al., 2018). Loss of BBB integrity following TBI increases vascular permeability and contributes to reduced cerebral blood and elevated vasogenic cerebral edema (Unterberg et al., 2004; Marmarou et al., 2006), and the latter is thought be a result of AQP protein dysfunction, which regulates the balance between preventing and facilitating water movement (Filippidis et al., 2016). In this study, we found that BL-HTWP treatment promoted cerebral blood perfusion restoration and improved performance in the MEP and mNSS tests after acute TBI. We also found this treatment reduced formation of gaps in tight junctions (Occludin and ZO-1) and expression of AQP4 and showed a protective effect on BBB integrity after TBI, implying that BL-HTWP plays a role in inhibition of vasogenic cerebral edema and may reduce the risk of cerebral hemorrhage by protecting the BBB by slowing the release of TF and thus retaining more coagulation factors.

A growing body of evidence has demonstrated that brain tissue/vessel injury, and even hypoperfusion, activated inflammation pathways triggered by endothelium activation after TBI and aggravated coagulopathy then occurred with extensive cross-talk between coagulation and inflammation pathways (Sillesen et al., 2014; Di Battista et al., 2016; Foley and Conway, 2016). Thrombin can act on microglia, cause cytoskeleton rearrangement of microglia, stimulate their proliferation and activation, and increase the synthesis of proinflammatory mediators such as IL-1β and IL-6 (Nimmerjahn et al., 2005; Schachtrup et al., 2010; Lee and Suk, 2017). Furthermore, these proinflammatory cytokines could lead to the upregulation of intercellular adhesion molecule-1 (ICAM-1) – a marker of endothelial damage – which mediated leukocyte transendothelial migration as part of a cascade of molecular interactions and associated with prolonged inflammation (Svetlov et al., 2012; Glushakova et al., 2014; Vignoli et al., 2018). Fibrinogen may affect the process of neuronal repair after trauma. It has been shown that fibrinogen inhibits neurite growth by activating the epidermal growth factor receptor in neurons and which promotes astroglial scar formation by acting as a potential carrier of transforming growth factor-β (Schachtrup et al., 2007; Schachtrup et al., 2010). Above these factors may cause the damage of the gliovascular unit and aggravate the cross-talk between coagulation and inflammation (Wolburg et al., 2009). In this study, we found larger numbers of GFAP^+^–astrocyte and Iba-1^+^–microglia, and higher levels of IL-6, IL-1β, and ICAM-1 in the peritraumatic areas of brain tissue after TBI than those of the sham control, which was in accord with previous studies. In contrast, fewer GFAP^+^- and Iba-1^+^-cells and lower levels of proinflammatory cytokines were mentioned above in the BL group than in the CCI mice, further suggesting that the function of BL-HTWP is associated with anti-inflammatory effect, which acts at least partly through the inhibition of coagulation.

In parallel, it has been generally assumed that molecular events post-TBI could be counterbalanced by two central processes that are involved in endogenous repair: trophic support and immunomodulation that mediate neuroinflammation (Tovar-y-Romo et al., 2016). Laboratory and clinical studies have demonstrated that trophic factors such as BDNF and VEGF are closely associated with the maintenance of normal function of the nervous system. Increased BDNF level can reduce excitotoxicity, microglial activation, and neuroinflammation (Jiang et al., 2011; Baydyuk et al., 2015). Additionally, the role of VEGF as an endogenous factor of recovery after CNS damage is proven in stroke, where ischemia induces the stabilization of HIF-1α as a transcriptional activator of VEGF (Lopez-Hernandez et al., 2012). In this study, we found low levels of BDNF and VEGF at different time points after TBI, whereas BL-HTWP significantly increased their expression levels. Intriguingly, the level of HIF-1α quickly increased at 6 h and then decreased rapidly from 24 to 72 h after TBI, but this low level was maintained over a broad period in the BL group, which showed an asynchronous state with VEGF level. These results suggested that BL-HTWP indirectly upregulates the VEGF expression via other upstream activators rather than HIF-1α. Further studies delineating the mechanism of these observed effects are warranted.

BL-HTWP has been applied as one of the first aid measures in various types of emergency for more than 3,000 years in China. Previous studies have found that it can improve the state of consciousness and increase systolic blood pressure in patients with severe craniocerebral trauma (Zhang et al., 2016). Other studies have shown that it reduces PT and aPTT time in patients with blood coagulation caused by stroke (Zhang et al., 2003). The molecular mechanism of BL-HTWP restoring the balance of coagulation system is not clear, but it may be related to maintaining cerebral microcirculation perfusion and protecting the structure and function of BBB (Li et al., 2017, 2019; Yu et al., 2017). To date, studies have shown that stimulating specific body surface sites can cause specific nuclei to secrete a variety of neurotransmitters; for example, a study activated the mesencephalic aqueduct to secrete orexin by stimulating Neiguan (PC6), which is located in the median nerve (Chen et al., 2018). Another study activated endorphin receptors in the hypothalamic arcuate nucleus by stimulating Shenmen (HT7), which is located at the distribution of the ulnar nerve in the wrist flexor tendon (Chang et al., 2019). Interestingly, we can see that the neurotransmitters activated by stimulating different acupoints are not exactly the same, which may be linked to the anatomical structure of the body surface where the acupoints are located. The position of the hand twelve *Jing-well* points is unique, all located at the end of the fingertips, and the fingertips have very rich sensory nerve endings and capillary network. From this, we can speculate that when using BL-HTWP treatment, first, the pain stimulus signal was uploaded to the nerve center after being amplified several times and activated certain specific nucleus to secrete certain types of neurotransmitters. To some extent, it plays a role in regulating vasoconstriction and stabilizing blood pressure. The second is to stimulate the vascular wall of the capillary network at the fingertips of the hand by bloodletting. The coagulation reaction is initiated locally and amplified through the cascade effect of the coagulation system, and then the coagulation disorder of the whole body is adjusted in a hedging manner. It is also worth noting that BL-HTWP is a treatment consisting of multiple stimuli. According to our analysis, there are at least three factors: bleeding, pain stimulation, and acupoint specificity. Studies have shown that no matter which of the above factors is excluded, although the amplitude of cerebral blood flow can be increased to a certain extent, its effect is significantly weakened than before (Zhou and Tp, 1998; Zhou et al., 1999). This also shows that BL-HTWP is a systematic regulation method, which may result in coordinated regulation of multiple targets.

**FIGURE 9:**
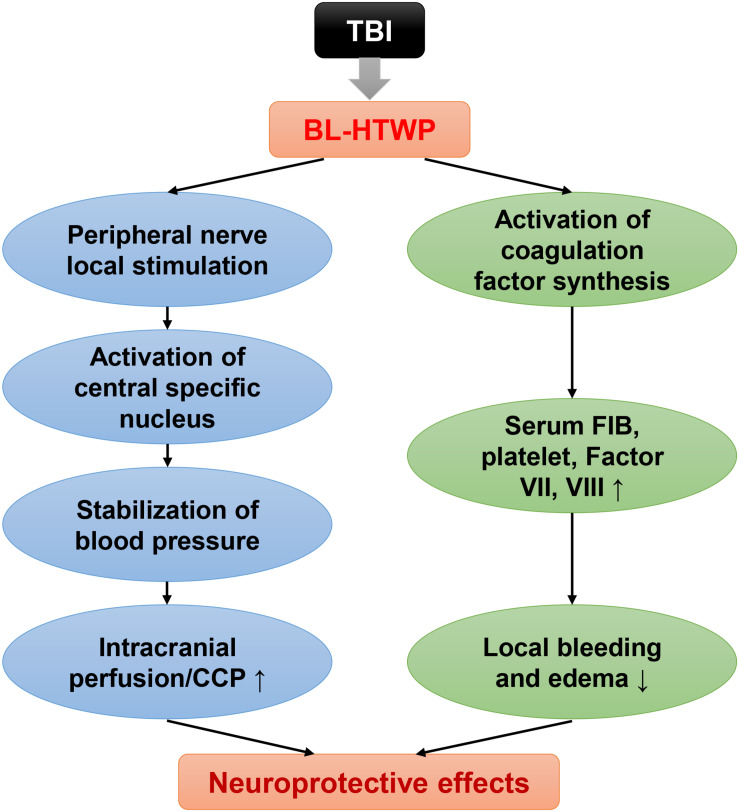
Mechanisms of neuroprotective effects of BL-HTWP post-TBI.

Taken together, these results demonstrated that coagulopathy is closely involved in the pathologic process of tissue/vessel injury, BBB disruption, and inflammatory response after acute TBI in mice. We conclude that bloodletting puncture at hand twelve *Jing*-well points improve neurological function after TBI in two ways (Figure 9). First, local stimulation of peripheral nerves after acupuncture causes central specific nucleus activation, which stabilizes or increases blood pressure and increases intracranial blood perfusion. Second, blood loss after bloodletting puncture causes the level of procoagulant factor synthesis, ameliorating local brain tissue hemorrhage and brain edema.

## Data Availability Statement

The datasets generated for this study are available on request to the corresponding author.

## Ethics Statement

The animal study was reviewed and approved by the Institutional Animal Care and Use Committee at Characteristic Medical Center of Chinese People’s Armed.

## Author Contributions

S-XC, BL, LZ, and SZ conceived and designed the experiments. BL, XZ, and T-LY conducted the experiments. Z-WX, YG, Y-MG, D-WP and Y-LC helped with the eperiments. S-XC and BL analyzed the data. BL and S-XC wrote the manuscript. All authors discussed and commented on the manuscript.

## Conflict of Interest

The authors declare that the research was conducted in the absence of any commercial or financial relationships that could be construed as a potential conflict of interest.
