# Supplementary material for: Bloodletting Puncture at Hand Twelve Jing-Well Points Improves Neurological Recovery by Ameliorating Acute Traumatic Brain Injury-Induced Coagulopathy in Mice
Source: Front Neurosci. 2020 Jun 5;14:403. doi: 10.3389/fnins.2020.00403 (PMC7290011; doi:10.3389/fnins.2020.00403)
Supplement: Supplementary file 1 [file Table_1.docx]

**Supplemental Material**

**Table S1 Sources and dilutions of the antibodies used in the study**

| **Antigen** | **Source** | **Catalog number** | **Dilution** | **Application** |
| --- | --- | --- | --- | --- |
| Occludin | abcam | ab167161 | 1:1000 | WB |
| ZO-1 | Proteintech | 63558 | 1:1000 | WB |
| AQP4 | abcam | ab46182 | 1:1000 | WB |
| BDNF | Bio-world | BS6533 | 1:1000 | WB |
| VEGF | Bio-world | BS6496 | 1:1000 | WB |
| TNF-α | Bio-world | BS6000 | 1:500 | WB |
| IL-6 | Bio-world | BS6419 | 1:500 | WB |
| IL-1β | Bio-world | BS6067 | 1:500 | WB |
| ICAM-1 | Bio-world | BS7138 | 1:1000 | WB |
| HIF-1alpha | abcam | ab179483 | 1:1000 | WB |
| GAPDH | Bio-world | MB001 | 1:1000 | WB |
| NeuN | Abcam | ab104225 | 1:500 | IF |
| GFAP | CST | #12389S | 1:200 | IF |
| Iba-1 | Proteintech | 10904-1-AP | 1:400 | IF |

CST, Cell Signaling Technology; WB,western blotting; IF, immunofluorescence

**Table S2 – Figure 2C Mean PU/mm^2^**

| **Group** | **Pre-** | **Time post-CCI (h)** | | | | |
| --- | --- | --- | --- | --- | --- | --- |
|  |  | **0** | **1** | **2** | **4** | **6** |
| **Sham** | 222.3±7.0 | 223.8±8.9 | 231.0±8.8 | 226.2±9.6 | 237.6±8.1 | 233.7±12.4 |
| **TBI** | 232.3±11.9 | 134.9±7.7 | 116.3±14.7 | 119.5±12.1 | 117.7±12.2 | 120.0±11.1 |
| **BL** | 223.9±14.5 | 143.9±18.1 | 180.4±16.8 | 183.0±25.6 | 179.8±21.6 | 179.5±23.0 |
| ***F*** | 0.915 | 70.652 | 92.145 | 56.947 | 87.102 | 58.296 |
| ***P*** | 0.439 | <0.001 | <0.001 | <0.001 | <0.001 | <0.001 |

**Table S3 – Figure 2D CPP**

| **Group** | **Pre-** | **Time post-CCI (h)** | | | | | | |
| --- | --- | --- | --- | --- | --- | --- | --- | --- |
|  |  | **0** | **2** | **4** | **6** | **12** | **18** | **24** |
| **Sham** | 76.22  ±0.47 | 75.71  ±1.71 | 75.04  ±1.84 | 77.42  ±1.10 | 75.28  ±1.96 | 75.57  ±1.44 | 73.92  ±1.26 | 74.09  ±1.61 |
| **TBI** | 77.42  ±1.58 | 25.25  ±1.87 | 28.38  ±1.60 | 35.19  ±1.71 | 36.73  ±1.71 | 40.28  ±0.84 | 45.22  ±2.22 | 50.18  ±2.85 |
| **BL** | 76.89  ±1.49 | 23.77  ±2.96 | 33.47  ±1.34 | 41.88  ±1.97 | 49.55  ±2.81 | 56.74  ±3.52 | 62.12  ±3.82 | 68.52  ±2.23 |
| ***F*** | 1.305 | 1033.248 | 1522.139 | 1156.161 | 473.151 | 368.982 | 177.214 | 179.572 |
| ***P*** | 0.3 | <0.001 | <0.001 | <0.001 | <0.001 | <0.001 | <0.001 | <0.001 |

**Table S4 –Figure 3B MEP latency**

| **Group** | **Latency LHL** | **Latency RHL** | **Amplitude LHL** | **Amplitude RHL** |
| --- | --- | --- | --- | --- |
| **Sham** | 5.20±0.12 | 5.18±0.13 | 2.39±0.09 | 2.15±0.55 |
| **TBI** | 7.13±1.10 | 5.78±0.58 | 1.19±0.26 | 2.20±0.75 |
| **BL** | 5.12±0.20 | 5.14±0.19 | 2.03±0.79 | 2.17±0.57 |
| ***F*** | 15.24 | 4.854 | 10.35 | 0.018 |
| ***P*** | <0.001 | 0.029 | 0.002 | 0.899 |

**Table S5 –Figure 3C mNSS score**

| **Group** | **Pre-** | **12 h** | **24 h** | **48 h** | **72 h** |
| --- | --- | --- | --- | --- | --- |
| **Sham** | 2.75±1.82 | 2.83±1.99 | 1.22±1.20 | 1.17±0.98 | 0.67±0.58 |
| **TBI** | 2.83±1.70 | 11.33±2.99 | 8.89±2.67 | 6.67±1.51 | 5.67±1.15 |
| **BL** | 2.58±1.68 | 8.83±1.80 | 5.67±1.50 | 5.67±0.82 | 5.33±0.58 |
| ***F*** | 0.81 | 42.454 | 37.028 | 39.615 | 35.167 |
| ***P*** | 0.52 | <0.001 | <0.001 | <0.001 | <0.001 |

**Table S6 –Figure 4A PT**

| **Group** | **PT** |
| --- | --- |
| **Sham** | 13.20±0.66 |
| **TBI** | 15.00±0.57 |
| **BL** | 13.44±0.68 |
| ***F*** | 11.620 |
| ***P*** | 0.002 |

**Table S7 –Figure 4B aPTT**

| **Group** | **aPTT** |
| --- | --- |
| **Sham** | 38.34±3.90 |
| **TBI** | 74.83±5.32 |
| **BL** | 45.50±7.17 |
| ***F*** | 59.296 |
| ***P*** | <0.001 |

**Table S8 –Figure 4C INR**

| **Group** | **INR** |
| --- | --- |
| **Sham** | 1.02±0.66 |
| **TBI** | 1.20±0.05 |
| **BL** | 1.04±0.07 |
| ***F*** | 13.016 |
| ***P*** | 0.001 |

**Table S9 –Figure 4D FIB**

| **Group** | **FIB** |
| --- | --- |
| **Sham** | 1.27±0.29 |
| **TBI** | 1.97±0.14 |
| **BL** | 1.63±0.08 |
| ***F*** | 16.974 |
| ***P*** | <0.001 |

**Table S10 –Figure 4E TT**

| **Group** | **TT** |
| --- | --- |
| **Sham** | 32.06±6.05 |
| **TBI** | 51.76±11.51 |
| **BL** | 45.36±5.96 |
| ***F*** | 7.404 |
| ***P*** | 0.008 |

**Table S11 –Figure 4F PTA**

| **Group** | **PTA** |
| --- | --- |
| **Sham** | 97.60±10.64 |
| **TBI** | 75.00±5.15 |
| **BL** | 94.60±9.66 |
| ***F*** | 9.695 |
| ***P*** | 0.003 |

**Table S12 –Figure 4G platelet count**

| **Group** | **Platelet** |
| --- | --- |
| **Sham** | 729.20±59.40 |
| **TBI** | 242.52±44.85 |
| **BL** | 602.66±58.82 |
| ***F*** | 106.267 |
| ***P*** | <0.001 |

**Table S13–Figure 4H Factor VII**

| **Group** | **Factor VII** |
| --- | --- |
| **Sham** | 96.38±7.66 |
| **TBI** | 51.10±3.62 |
| **BL** | 85.23±4.51 |
| ***F*** | 90.547 |
| ***P*** | <0.001 |

**Table S14 –Figure 4I Factor VIII**

| **Group** | **Factor VIII** |
| --- | --- |
| **Sham** | 182.28±16.82 |
| **TBI** | 124.08±18.18 |
| **BL** | 173.38±17.65 |
| ***F*** | 15.941 |
| ***P*** | <0.001 |

**Table S15 –Figure 5B R time**

| **Group** | **R** |
| --- | --- |
| **Sham** | 2.76±0.29 |
| **TBI** | 3.96±0.68 |
| **BL** | 3.34±0.23 |
| ***F*** | 9.018 |
| ***P*** | 0.004 |

**Table S16 –Figure 5C K time**

| **Group** | **K** |
| --- | --- |
| **Sham** | 1.3±0.43 |
| **TBI** | 3.24±0.59 |
| **BL** | 2.54±0.27 |
| ***F*** | 23.696 |
| ***P*** | <0.001 |

**Table S17 –Figure 5D MA value**

| **Group** | **MA** |
| --- | --- |
| **Sham** | 64.14±1.69 |
| **TBI** | 72.58±3.91 |
| **BL** | 63.08±3.74 |
| ***F*** | 12.628 |
| ***P*** | <0.001 |

**Table S18 –Figure 5E angle**

| **Group** | **angle** |
| --- | --- |
| **Sham** | 72.16±4.90 |
| **TBI** | 55.46±2.80 |
| **BL** | 68.06±6.26 |
| ***F*** | 15.979 |
| ***P*** | 0.001 |

**Table S19 –Figure 6C Evans blue extravasation**

| **Group** | **EB** |
| --- | --- |
| **Sham** | 2.60±0.27 |
| **TBI** | 7.51±1.10 |
| **BL** | 4.79±0.82 |
| ***F*** | 37.052 |
| ***P*** | <0.001 |

**Table S20 –Figure 6D Brain water content**

| **Group** | **%** |
| --- | --- |
| **Sham** | 77.01±1.36 |
| **TBI** | 78.47±2.33 |
| **BL** | 75.90±1.79 |
| ***F*** | 5.121 |
| ***P*** | 0.023 |

**Table S21 –Figure 7D Percentage of NeuN + -, GFAP + -, and Iba-1 + -cells**

| **Group** | **NeuN** | **GFAP** | **Iba-1** |
| --- | --- | --- | --- |
| **Sham** | 79.69±3.88 | 4.43±0.85 | 6.32±0.92 |
| **TBI** | 38.77±4.03 | 18.06±4.20 | 17.78±2.33 |
| **BL** | 52.23±3.10 | 7.85±0.82 | 11.42±2.96 |
| ***F*** | 127.262 | 31.657 | 26.280 |
| ***P*** | <0.001 | <0.001 | <0.001 |

**Table S22 –Figure 8B AQP4**

| **Group** | **6 h** | **24 h** | **48 h** | **72 h** |
| --- | --- | --- | --- | --- |
| **Sham** | 0.16±0.01 | 0.16±0.01 | 0.16±0.01 | 0.16±0.01 |
| **TBI** | 1.28±0.11 | 0.57±0.02 | 0.67±0.03 | 0.90±0.05 |
| **BL** | 1.17±0.05 | 0.69±0.03 | 0.28±0.01 | 0.65±0.02 |
| ***F*** | 355.750 | 645.078 | 1082.158 | 494.043 |
| ***P*** | <0.001 | <0.001 | <0.001 | <0.001 |

**Table S22 –Figure 8B Occludin**

| **Group** | **6 h** | **24 h** | **48 h** | **72 h** |
| --- | --- | --- | --- | --- |
| **Sham** | 0.28±0.02 | 0.28±0.02 | 0.28±0.02 | 0.28±0.02 |
| **TBI** | 0.11±0.01 | 0.15±0.01 | 0.11±0.02 | 0.15±0.01 |
| **BL** | 0.29±0.01 | 0.07±0.01 | 0.73±0.02 | 0.71±0.02 |
| ***F*** | 321.215 | 492.481 | 1747.718 | 1031.060 |
| ***P*** | <0.001 | <0.001 | <0.001 | <0.001 |

**Table S23 –Figure 8B ZO-1**

| **Group** | **6 h** | **24 h** | **48 h** | **72 h** |
| --- | --- | --- | --- | --- |
| **Sham** | 0.52±0.02 | 0.52±0.02 | 0.52±0.02 | 0.52±0.02 |
| **TBI** | 0.31±0.02 | 0.16±0.01 | 0.17±0.01 | 0.19±0.01 |
| **BL** | 1.45±0.07 | 0.90±0.02 | 0.70±0.05 | 0.78±0.03 |
| ***F*** | 737.042 | 1345.839 | 319.884 | 702.246 |
| ***P*** | <0.001 | <0.001 | <0.001 | <0.001 |

**Table S24 –Figure 8B IL-6**

| **Group** | **6 h** | **24 h** | **48 h** | **72 h** |
| --- | --- | --- | --- | --- |
| **Sham** | 0.45±0.01 | 0.45±0.01 | 0.45±0.01 | 0.45±0.01 |
| **TBI** | 0.68±0.04 | 0.36±0.01 | 0.74±0.01 | 0.66±0.01 |
| **BL** | 0.09±0.01 | 0.03±0.01 | 0.40±0.02 | 0.59±0.03 |
| ***F*** | 676.056 | 2633.102 | 912.402 | 141.081 |
| ***P*** | <0.001 | <0.001 | <0.001 | <0.001 |

**Table S25 –Figure 8B IL-β**

| **Group** | **6 h** | **24 h** | **48 h** | **72 h** |
| --- | --- | --- | --- | --- |
| **Sham** | 0.34±0.02 | 0.34±0.02 | 0.34±0.02 | 0.34±0.02 |
| **TBI** | 0.70±0.02 | 0.58±0.04 | 0.90±0.04 | 0.74±0.05 |
| **BL** | 0.57±0.01 | 0.13±0.02 | 0.25±0.02 | 0.05±0.02 |
| ***F*** | 327.019 | 344.362 | 520.375 | 317.442 |
| ***P*** | <0.001 | <0.001 | <0.001 | <0.001 |

**Table S26 –Figure 8B ICAM-1**

| **Group** | **6 h** | **24 h** | **48 h** | **72 h** |
| --- | --- | --- | --- | --- |
| **Sham** | 0.21±0.02 | 0.21±0.02 | 0.21±0.02 | 0.21±0.02 |
| **TBI** | 0.55±0.02 | 0.44±0.01 | 0.35±0.02 | 0.67±0.03 |
| **BL** | 0.27±0.01 | 0.10±0.01 | 0.44±0.01 | 0.36±0.02 |
| ***F*** | 322.474 | 598.664 | 159.026 | 284.295 |
| ***P*** | <0.001 | <0.001 | <0.001 | <0.001 |

**Table S27 –Figure 8B BDNF**

| **Group** | **6 h** | **24 h** | **48 h** | **72 h** |
| --- | --- | --- | --- | --- |
| **Sham** | 1.79±0.07 | 1.79±0.07 | 1.79±0.07 | 1.79±0.07 |
| **TBI** | 0.12±0.01 | 0.05±0.01 | 0.29±0.01 | 0.22±0.01 |
| **BL** | 1.07±0.02 | 0.61±0.01 | 0.64±0.01 | 0.36±0.03 |
| ***F*** | 1488.474 | 1698.276 | 1351.663 | 1541.583 |
| ***P*** | <0.001 | <0.001 | <0.001 | <0.001 |

**Table S28 –Figure 8B VEGF**

| **Group** | **6 h** | **24 h** | **48 h** | **72 h** |
| --- | --- | --- | --- | --- |
| **Sham** | 0.13±0.01 | 0.13±0.01 | 0.13±0.01 | 0.13±0.01 |
| **TBI** | 0.04±0.01 | 0.03±0.01 | 0.05±0.01 | 0.10±0.01 |
| **BL** | 0.68±0.01 | 0.56±0.03 | 0.65±0.02 | 0.59±0.02 |
| ***F*** | 1296.384 | 1598.166 | 1571.568 | 1569.453 |
| ***P*** | <0.001 | <0.001 | <0.001 | <0.001 |

**Table S29 –Figure 8B HIF-1α**

| **Group** | **6 h** | **24 h** | **48 h** | **72 h** |
| --- | --- | --- | --- | --- |
| **Sham** | 0.27±0.01 | 0.27±0.01 | 0.27±0.01 | 0.27±0.01 |
| **TBI** | 0.72±0.03 | 0.14±0.01 | 0.05±0.01 | 0.08±0.01 |
| **BL** | 0.03±0.01 | 0.04±0.01 | 0.03±0.01 | 0.02±0.01 |
| ***F*** | 1245.345 | 2904.166 | 1612.568 | 1435.453 |
| ***P*** | <0.001 | <0.001 | <0.001 | <0.001 |
